# Rethinking posthumanism in rehabilitation science: Lessons from Indigenous, Black, and decolonial thought

**DOI:** 10.1177/13634593251345082

**Published:** 2025-06-13

**Authors:** Eduan Breedt, Erin Tichenor, Kim McLeod, Tim Barlott

**Affiliations:** University of Alberta, Canada; University of Alberta, Canada; The University of Tasmania, Australia; University of Alberta, Canada

**Keywords:** colonialism, healthcare, posthumanism, racism, rehabilitation science

## Abstract

Posthumanism is a theoretical paradigm in Western continental philosophy with emerging significance and popularity in the health disciplines. Rehabilitation science scholars in fields like occupational therapy and physical therapy have taken up posthumanism, valuing its interventions into the harms of European humanist conceptualizations of the “(hu)man” which perpetuate individualism, ableism, and anthropocentrism. This paper responds to the pervasive use of posthumanism in the rehabilitation science literature—particularly among white scholars in the “Global North”—and its omission of sustained engagements with forms of *de*humanization (specifically racism, colonialism, and anti-Blackness). For posthuman healthcare and rehabilitation scholarship to have utility beyond white, globally elite populations, we invite fellow rehabilitation science scholars to engage with the important critiques of posthumanism made by Black, Indigenous, and Latin American decolonial scholars. We synthesize these critiques and warnings about the forms of epistemic colonial violence embedded within popular approaches to posthumanism, and query rehabilitation scholars’ responsibilities to pause and center theories of the human and posthuman that have long been developed and lived by racialized and Indigenous scholars, activists, and knowledge holders.

## Introduction

Posthumanism is a theoretical framework and field of study concerned with how “man” and “human” have been defined, conceptualized, and operationalized within Western continental philosophy. Posthumanism emphasizes the ecological and social harms that have come from anthropocentric Enlightenment philosophies that draw marked distinctions between humans and non-humans (Bignall and Rigney, 2018; Mignolo and Walsh, 2018). Many posthuman theorists critique European humanism for using these frameworks to categorize different groups of people as “fully human,” “sub-human,” and “non-human.” Several social theorists, however, have critiqued posthumanist philosophy—its foundational assumptions as well as how it is applied—for focusing on ontological arguments about non-human matter and overlooking its own implication in racializing and colonial logics (Byrd, 2011; Jackson, 2013, 2015; Karera, 2019; King, 2017; Mignolo, 2012; Mignolo and Walsh, 2018; Moten, 2013; Todd, 2016; Weheliye, 2014).

Despite these critiques, posthumanism is an increasingly popular framework being taken up in the applied health and social sciences. This paper is specifically directed toward rehabilitation science scholars using—or interested in using—posthumanism. Rehabilitation science is the school of thought behind rehabilitation medicine, a multidisciplinary field dedicated to supporting individuals experiencing acute and chronic illness, injury, and disability. Rehabilitation clinicians (e.g. physical therapists, occupational therapists, speech-language pathologists) focus on restoring and enhancing individuals’ functional abilities so that they can engage in their activities of daily living and participate in their communities and society more broadly (Gutenbrunner et al., 2011; Meyer et al., 2011; Stucki et al., 2018; World Health Organization, 2025). Rehabilitation science supports these efforts by drawing on social science, health science, and engineering to analyze the social, environmental, technological, behavioral, and health system contexts that shape clinical and institutional approaches to preventing, treating, and managing disabling health conditions and experiences (World Health Organization, 2025).

Scholars in rehabilitation disciplines such as occupational therapy, physiotherapy, and speech-language pathology (as well as those outside of rehabilitation, such as disability studies and nursing) are drawing on posthumanist theories to address the social, environmental, and theoretical contexts of health and rehabilitation. A cursory Google Scholar search reveals that published articles on posthumanism in rehabilitation science have almost doubled between 2019 and 2024, rendering it “one of the most vibrant fields in healthcare philosophy and sociology today” (Nicholls, 2024a: n.p; see also Nicholls, 2021; Nicholls, 2023; Nicholls et al., 2023). Within the posthumanist rehabilitation literature, when we searched for the terms such as “race,” “racism,” and “colonial,” we retrieved almost no results. When these terms were present, there was no substantial engagement with these matters. This finding may indicate that rehabilitation science scholars—including ourselves—*are not* engaging with posthumanist theory and commentary beyond the work of European and North American philosophers, nor with posthumanist work in other disciplines that situate anti-racism and anti-colonialism as central to their posthumanist endeavors (see Bignall, 2022; Bignall and Rigney, 2018; Brown et al., 2022; Chen, 2012; Collins, 2023; Ellis, 2018; Hopkins-Walsh et al., 2022; Ikemoto, 2005; Islam, 2016; Jackson, 2018, 2020; McLeod and Fullagar, 2021; Polsky, 2022; Puar, 2017; Romero et al., 2023; Rosiek et al., 2020; Smith et al., 2022; Sundberg, 2014; Van Patter et al., 2023; Weheliye, 2014).

A noteworthy stimulus for writing this paper was our participation at the 2024 In Sickness and In Health Conference, which serves as a platform for “radical, cutting-edge critical theories and philosophies” (In Sickness and In Health, 2024) in the healthcare disciplines. The 2024 conference exhibited several excellent presentations that specifically attended to decolonizing and Indigenizing health and well-being (Bernheim, 2024; Blanchet Garneau et al., 2024; Bourque Bearskin et al., 2024; Haami, 2024; Neely et al., 2024; Ngaropo, 2024; Stewart, 2024). However, what was also particularly noteworthy was the number of presentations using posthuman theory in healthcare scholarship. Aside from McLeod’s (2024) presentation, “How white is more-than-human health research?,” we noticed that many of the presentations using posthumanism did not explicitly address the specific issue of European humanism, its racial and colonial consequences, and theories of the human that have long critiqued and existed outside of European humanism. This observation is not intended to distance ourselves from these oversights—two of us have written about posthumanism in ways that have replicated these very omissions (Barlott and Setchell, 2023; McLeod, 2017), and all four of us have been (or still are) drawn to its potential. Posthumanism’s appeal, with its provocations against human exceptionalism and its potential for rethinking bodies and relations, is undeniably compelling. Yet, at the conference, the convergence of our recent engagements with critical theorists, the charged political moment, the conference’s specific context and makeup, our diverse experiences across Canada, Aotearoa New Zealand, Australia, and South Africa, and a range of unnamed factors, brought these absences into sharper relief and made them deeply unsettling. We found ourselves drawn together at the conference, connecting over a shared discomfort and desire to reckon with the ways in which our own engagement with posthumanism has overlooked or obscured its entanglements with racism, colonialism, and European humanism’s ongoing violent legacy.

In light of the seemingly growing popularity of posthumanism among rehabilitation scholars, particularly white academics in the “Global North” like us, what questions does this raise about the theory’s utility beyond globally privileged academics and communities?

This article does not offer an in-depth analysis of, or solution to, specific issues in the posthumanist rehabilitation science literature. Rather, this conference experience and our observations of the rehabilitation science literature have compelled us to further engage with the key critiques of posthumanism made by scholars in Critical Black Studies, Indigenous and Settler Colonial Studies, Critical Race Theory, and African and Latin American Decolonial theory—and to bring these critiques to the forefront of rehabilitation science. That is, given the limited analyses we hold as white settler researchers, we do not ourselves address the complexity of debates occurring within and outside of posthumanist philosophy ourselves, but invite fellow white, elite rehabilitation scholars to join us in engaging in self-critique and reflecting on the allure of posthumanism, the harms it might be obscuring and perpetuating, and who and what systems it might be serving.

As we have alluded to, the four of us are white settler scholars from South Africa, the United States, Australia, and Canada, and our authorship on this paper about epistemic violence raises several ethical dilemmas. Our considerations about these conundrums are ongoing and unfinished. We would first like to make clear that we are implicated in the aforementioned trends in health and rehabilitation science. We (the first two authors) have been attracted to and drawn on aspects of posthumanism and other well-critiqued Western frameworks such as new materialism in our graduate work. We (the third and fourth authors) have written books and articles engaging with posthumanism within health sociology and rehabilitation science. Second, we acknowledge that we are perpetuating the critiques we lay out in this paper by acting as “white intermediaries” and “filtering” the ideas of the racialized and Indigenous scholars who have made these interventions (Todd, 2016: 7). Finally, we benefit professionally from the publication of this paper, as well as through its recentering of white theorists writing on posthumanism. Considering these issues, we put forth a few circumstances and rationales surrounding our decision to come together as authors. The first is the context within which this article emerged. We (the first two authors) are graduate students supervised by the fourth author. Partway through our programs, we became concerned with the proliferation of Western theories that may not be as liberatory as they seem (e.g. new materialism and critical disability studies). We connected with the third author, who has expertise in this topic, while attending the In Sickness and In Health conference. Notably, we do not know racialized scholars in our spheres that explicitly write about posthumanism, and thus did not consult or collaborate with racialized authors. This raises questions about our own relations, the limits of our own positionality, as well as the relative absence of racialized scholars in health-related spheres writing about posthumanism.

To the contrary, however, we recognize that the burden of anti-racist work is frequently placed on racialized scholars despite many having long insisted that white scholars and activists take responsibility for it (Dabiri, 2021; Eddo-Lodge, 2017; Oluo, 2018; Reid, 2022; Saad, 2020). We thus treat this introductory article as a response to the calls for us to talk among ourselves, speak back to whiteness, and take responsibility for addressing the issue of white supremacy and colonial epistemicide among white, Western scholars.

All that can save you now is your confrontation with your own history [. . .] which is not your past, but your present—Baldwin (1968: n.p).

We urge readers to practice a citational politics that decenters whiteness and foregrounds those who are consistently underrepresented in citational lineages (Ahmed, 2014). For example, rather than only citing this article, read and cite the work of racialized and Indigenous authors instead.

In what follows, we briefly introduce posthumanism and its value in rehabilitation scholarship. We then foreground the work of Indigenous, decolonial, and critical race scholars who have critiqued the problematic presuppositions in much of posthumanist thought. To conclude, we invite readers to consider how—and if—rehabilitation science, as a predominantly white institution, engages with posthumanism so as to avoid perpetuating the growing problem of silence about racism and colonialism within rehabilitation science scholarship.

## Utility of posthumanism in rehabilitation science

Posthumanism has diverse lineages and can be understood as both a theoretical framework (Braidotti, 2013) and a historical condition that emerged from the ideological trajectories of postmodernism and neoliberalism (Hayles, 1999). When posthumanism was first coined by Ihab Hassan in his 1977 essay *“Prometheus as Performer: Toward a Posthumanist Culture?,”* he was analyzing humanity’s ongoing transition into a posthuman condition. His was an analysis of this cultural and technological shift. Similarly, N. Katherine Hayles (1999) and more recently, Stephanie Polsky (2022) have explored how cybernetics and virtual bodies have reshaped what it means to be human. These preeminent posthumanist scholars have been mapping and critiquing the ways that humanity is already situated within a posthuman culture—whether we like it or not—and the consequences of this shift (Jansen et al., 2021).

Within Western continental philosophy, however, posthumanism can largely be understood as a Eurocentric response to, and critique of, the problems of European modernism, such as the social and ecological injustices that have come from European humanist conceptualizations of “man” and its resulting anthropocentrism. For example, scholars such as Rosi Braidotti (2013), Jane Bennett (2010), Donna Haraway (1987), Karen Barad (2007), and Claire Colebrook (2014) challenge European humanism’s categorization and prioritization of some phenomena over others, such as rationalism over emotion, mind over body, and human over non-human—to name a few (Bignall and Rigney, 2018; Mignolo and Walsh, 2018). Since “all matter matters,” posthumanism exposes these exclusions and hierarchies embedded within European conceptualizations of the (hu)man, which sparked the colonial practices of rendering colonized people to “sub-human” and “non-human” categories (Barad, 2007; Bignall, 2022; Braidotti, 2013). Braidotti (2019: 54) is careful not to slip into relativism, suggesting that “[t]he proper subject of the posthuman convergence is not ‘Man’, but a new collective subject, a ‘we-are-(all)-in-this-together-but-we-are-not-one-and-the-same’ kind of subject.” Braidotti (2018: 35) continues, stating that the “human” “never was a universal or a neutral term to begin with. It is rather a normative category that indexes access to privileges and entitlements.” Barad (2007: 20) similarly focuses on “how differences get made, what gets excluded and how those exclusions matter.” In response to these dehumanizing hierarchies, posthumanist philosophers have reasserted the shared ontological value, agency, vulnerability, among, not only humans, but human and non-human matter. This intervention challenges transcendent European ontologies that place certain humans as superior to and separate from the rest of the world, and cuts through the anthropocentric hubris that has put so much human and non-human life at risk. As a whole, many posthumanists have thus urgently called for a “move beyond the human” and a “turn to more-than-human matter” so that our scholarship can better appreciate the ontological agency possessed by all matter and situate humans within a broader world, wherein we are in constant interaction with organic and inorganic matter. This call is the predominant one associated with critical posthumanism, and the one most taken up by rehabilitation scholars developing “more-than-human” analyses of health and healthcare.

Posthumanist philosophy has become increasingly popular in healthcare research because of its ability to (1) attend to the myriad of co-constitutive material, social, environmental, technological, informatic, and affective forces that produce health, ill-health, and health inequality, and (2) expose how individualistic and human-centered approaches to health cannot attend to broader ecological and cosmological systems—systems that are under threat due to this anthropocentrism. That is, health scholars argue that posthumanism may better equip healthcare scholars and clinicians to address the climate crisis *and* its impact on human and non-human health (see Andrews and Duff, 2019; Bortolotti et al., 2022; Bozalek and Pease, 2020; Cohn and Lynch, 2017; Collins, 2023; Fox, 2024; Friese and Nuyts, 2017; Hopkins-Walsh et al., 2022; Laing, 2020; Laurin and Martin, 2024; McLeod and Fullagar, 2021, 2025; McPhie, 2019; Petrovskaya, 2023; Richards, 2023; Rock, 2017; Tanioka et al., 2021; Thorne, 2024; Woodlan, 2016).

Rehabilitation science is no exception to this trend and has taken up posthumanism to support researchers and clinicians in accounting for the variety of non-human factors that can lead to a particular health outcome, beyond the often blamed and pathologized individual (Gibson et al., 2021). These shifts have had implications for both the theory and practice of rehabilitation medicine. For example, posthumanism can aid in the reconceptualization of “care” as an emergent entanglement of social and material relations, not simply a unidirectional transaction between care provider and care recipient (see Barlott and Setchell, 2023; Cozza et al., 2021; Gibson et al., 2021). Care, in this sense, is not limited to the biomechanical or biochemical intervention, but to the equally important verbal and nonverbal, affective, embodied, cultural, socio-political, and environmental aspects of the care encounter (see Abrams et al., 2019; Fadyl et al., 2020; Gibson et al., 2021; Mescouto, 2023; Setchell et al., 2021). The human is no longer understood as an autonomous, discrete, static, Cartesian thing, separate from and superior to the world, but, as Gibson (2006: 195) notes, as a radically open assemblage of continual “flowing connections” between matter (biological, social, chemical, and physical; see also Holmes et al., 2024; Mescouto, 2023; Nicholls and Gibson, 2010). A wheelchair, for instance, is not merely “an ‘aid’ to a person’s mobility” but “a necessary and equal partner in an assemblage that allows a person to move” (Nicholls, 2018: 110). In deconstructing how Enlightenment assumptions about “man” have contributed to the pathologization of non-normative bodies, posthumanist theory has also contributed to critical disability perspectives, both within and beyond rehabilitation science (see Gibson et al., 2021, 2025; Gibson and Mosleh, 2024; Goodley et al., 2014; Grazyna, 2020; Liddiard et al., 2019; Michalko and Goodley, 2023; Waterworth et al., 2020). Posthumanism’s decentering of the human, human identity, voice, and individual desires has also helped rehabilitation scholars engage with the urgent topic of planetary health, and its relationship to human health (Maric and Nicholls, 2022). Nicholls (2018: 110) writes that “[o]xygen, processes of diffusion, muscle contractions and feelings of breathlessness, might be allied to air quality, pollution standards, and plant photosynthesis in an ontological equivalency that spans all human and non-human entities,” thus advocating for a respiratory therapy that conceptualizes itself as implicated in the climate crisis and its potential solutions. Finally, Turcotte et al. (2024: 1) have used posthumanism to resist neoliberal healthcare contexts and their constraints by urging professionals to embody a posthuman and “more-than-professional approach” that forge new social relations within healthcare settings, straying from conventional, apolitical notions of “professionalism.” These varied uses of posthumanism in rehabilitation science supports scholars and clinicians to look beyond the individual human, and grapple with all of the matter that affects their wellbeing and that they in turn affect.

Despite the recent progress made in recognizing the harms of European humanism and turning toward matter, to our knowledge, neither us nor our contemporaries in rehabilitation science using posthumanism have a sustained engagement with matters of race, racism, and colonialism, and their implications for illness, disease, injury, and death. The problems with these omissions become more stark when we consider the historical and ongoing role that colonial healthcare professions and systems play in Euro-American racism and colonialism (Anderson, 2006; Boodman, 2023; Downs, 2021; Fanon, 1965; Geddes, 2017; Lux, 2016; Mayes, 2020; Nuriddin et al., 2020; Paugh, 2017; Ratangee, 2023; Shaheen-Hussain, 2020; Sowemimo, 2023; Waitzkin, 2011; Washington, 2006). In what follows, we foreground the critiques of scholars who have argued that we are not simply seeing a citational shortcoming, but a fundamental flaw in several aspects of posthumanism.

## Critiques of posthumanism

Before moving onto the main critiques informed by critical race scholars, we would first like to note that there is an eerie mirroring of the political economy of late stage neoliberal capitalism in posthumanist scholarship, where both focus on unencumbered potentials, free-flowing boundaries, and seamless exchange between interconnected matter (Braidotti, 2013; Culp, 2016; Galloway, 2013; Hardt and Negri, 2000; Ibrahim, 2021; Nealon, 2021; Puar, 2017). As Braidotti (2013: 45) warns, “the advocates of advanced capitalism seem to be faster in grasping the creative potential of the posthuman than some of the well-meaning and progressive neo-humanist opponents of this system.” This may be unsurprising if, like Hassan (1977), Hayles (1999), and Polsky (2022), we remember that posthumanism was first analyzed as an era—something we have “become” in the wake of postmodernity and neoliberalism—and secondarily as a theoretical framework—something that we ought to “turn to.” Some posthuman rehabilitation scholars have recognized the possible confluence between “more-than-human” perspectives and neoliberal capital accumulation (Nicholls, 2024b), and while it is tempting to expand upon these biopolitical critiques, we are wary that a focus on how economic value is extracted from life cannot explain, and in fact, can obscure state-sponsored investments in gratuitous racial violence, death, and debility (Puar, 2017; Wang, 2012; Wilderson, 2008, 2020). An analysis of the political economy that minimizes direct relations of force or violence and “fails to address anti-Blackness, or only addresses it as a by-product of capitalism” is inadequate, and according to Jackie Wang (2012: n.p.) can only be theorized from a white subject position.

Our attempt to synthesize the following critiques of posthumanism is thus mainly centered around Zaikyyah Iman Jackson’s (2015: 215) question, “what and crucially whose conception of humanity are we moving beyond” (see also Burton, 2017; King, 2017; Weheliye, 2014)? As many Critical Black Studies and Indigenous scholars have been quick to highlight, in its desire to move beyond humanism, posthumanism has paradoxically recentered the concept of the human “rooted in the epistemological locus of the West” (Jackson, 2013: 672), without acknowledging the geopolitical and philosophical specificity of that humanism (even while it has had global effects; see also Bignall and Rigney, 2018). Euro-American scholars announced that they/we have come up with their/our own “profound” concept of the human, where “whiteness-as-humanness” is maintained (Sullivan, 2012: 303), while glossing over “alternative humanisms that have always pulled away from, pushed against, or existed with complete apathy for liberal humanism” (Burton, 2017: 19; see also Botha et al., 2021; De La Cadena, 2010; Jackson, 2015; King, 2017; Todd, 2016; Weheliye, 2014; Wynter, 1984, 2003; for scholarship that has situated posthumanism within or adjacent to non-Western philosophy, see Pang, 2022; Zembylas, 2018; Zhao, 2020).

In examining this recentering of Eurocentric problems and solutions, we first want to highlight the critiques made by several Indigenous scholars, who have noted posthumanism’s stark omission of Indigenous ontologies and cosmologies even though “for hundreds and thousands of years, interconnectedness [. . .] has been the mainstay in many Indigenous frameworks, both tribal and diasporic” (Tuck, 2010: 646). These worldviews, in their diverse ways, situate the human as amongst—not superior—to the natural world; however, given the frequent positioning of Indigenous thought as “preconceptual” (prior to “structured thought systems”; Byrd, 2011: 112), it is perhaps unsurprising that Western posthumanism has emerged from a recent “breathless realization” (Todd, 2016: 16) that we are interconnected with the natural world. In other words, posthumanism has “coloniz[ed] rather than validat[ed] Indigenous worldviews and ways of thinking, doing and being” (McLeod, 2024, n.p.) by “spinning itself on the backs of non-European thinkers” (Todd, 2016: 7). Métis scholar Zoe Todd (2016: 18) reiterates that “[w]hen we cite European thinkers who discuss the ‘more-than-human’ but do not discuss their Indigenous contemporaries who are writing on the exact same topics, we perpetuate the white supremacy of the academy” and the exploitation of Indigenous peoples.

While noting this appropriation, we also highlight the remarks that integrating human/nature or culture/nature is nonsensical to Indigenous thought, because, as Chickasaw theorist Jodi Byrd (2011) notes, these concepts have never been separate in Indigenous ontologies. Alfred López (2018: 380) reiterates that “nature-culture dualism [. . .] is itself the product of intra-rather than inter-action: an intra-Western dialogue (and thus really, culturally speaking, a monologue), a masturbatory f(r)iction that only collaterally, instrumentally rubs up against a real Other, even then still mistaking it for a thing.” Furthermore, Byrd reminds us that humans *do* have specific agency and responsibility in much of Indigenous thought, in contrast to the posthumanist de-responsibilization of the human. Byrd observes that the positioning of the posthuman subject as devoid of agency enables a sustained insensitivity to Indigenous dispossession of land. African decolonial scholars Nhemachena and Mawere (2020: 16) echo this, highlighting that when posthumanists regard humans as ontologically indistinct from nonhumans, “Indigenous people are urged to simply adapt and be resilient to security crises that are imperially generated—they are never urged to respond to the crises by repossessing or reclaiming their land and other resources stolen during the colonial era.” In sum, while posthumanism appropriates aspects of Indigenous worldviews, it botches and overlooks many critical aspects.

Alexander Weheliye (2014: 10) reminds us that not only has posthumanism assumed that it is European humanism that “we all want to overcome,” but that “all subjects have been granted equal access to Western humanity.” Karera (2019), Mignolo and Walsh (2018), and Todd (2016) similarly argue that by centering one specific type of humanism that we need to go beyond, posthumanism offers itself as a universal solution to a provincial problem. In doing so, she argues, posthumanism has a tendency to render us all equally *vulnerable to* and *responsible for* the apocalyptic consequences of anthropocentrism—the implication being that we should band together in an interspecies alliance. Even the most critical of posthumanist thought tends to end with universalizing “pan-human” approaches that bypass the production and distribution of both racial violence *and* climate disaster, a phenomenon that is itself racially patterned in its consequences.

On *vulnerability*, Axelle Karera (2019: 30, 32, 41) responds to scholars like Braidotti (2013: 100–101) and her suggestion that by recognizing our “common ground,” a common “we,” or “pan-humanity” we actualize a “community that is not bound negatively by shared vulnerability, the guilt of ancestral communal violence, or the melancholia of unpayable ontological debts, but rather by the compassionate acknowledgement of their interdependence with multiple others.” This naturalized and affirmative notion of interspecies relationality and interdependence deems death and debility as “‘common denominator’[s] for a ‘pan-human’ and inter-species alliance” (Karera, 2019: 42, referencing Braidotti, 2013: 111). Though Braidotti (2016: 23) herself later argues against this pan-human approach, the consistent return to an “affirmative politics” of a “shared understanding of what it means to be posthuman” can, as Diana Leong (2016: 11) suggests, “flatten the distinctions between traumas inflicted through happenstance and persistent intergenerational harm.” In other words, Karera and Leong argue that Braidotti’s (2007, 2009, 2013) own commentary on necropolitics and the uneven distribution of death gets smoothed over by her’s (and others’) emphasis on the survival of a pan-humanity. Braidotti’s (2011) invocation of “living with our wounds” as the basis for an affirmative politics of life, may, as Jasbir Puar (2017: 65) suggests about white liberal appeals to futurity, only be tenable by those who perceive a wound “as the result of an unfortunate accident, or a misfortune, as an exceptional circumstance,” rather than as the direct and calculated result of the “exploitative capital and imperial structures of the global north” that target specific populations “deemed available for injury” (Puar, 2017: 64; see Livingston, 2005; Meekosha, 2011). Puar (in CLAGS: The Center for LGBTQ Studies, 2013) suggests that universalizing approaches to vulnerability or precarity, while perhaps ontologically valuable, can have the effect of diminishing bio- and necro-political forces and “real social antagonisms,” especially “those enacted along racial lines” (Karera, 2019: 43). Karera (2019: 39) shows how this flattening of the ways in which “we are all” imminently endangered, posthumanist theories can specifically sidestep the racial histories and racial productions of our current predicament and obscure the fact that we are not all, as Wang (2018: n.p.) reiterates, “equally fucked.” This universalizing appeal to our mutual abjections ignores the emergency that racialized, colonized, and Black populations have faced since colonization. In calling for us to urgently take up posthumanism before we all suffer the consequences, posthumanism arguably reveals itself as ill-equipped to grapple with racial violence, the creation of some humans as disposable, and the responsibility that certain humans have in our current crises of health inequity and climate disaster.

With regards to *responsibility*, entrenched in posthumanism—its foundations, as well as how it is popularly taken up—seems to be an implicit desire to forget the responsibility such violent colonial histories bestows upon the Western world. By conflating humanism and anthropocentrism with *all humans* and *all human worldviews*, as discussed, many posthumanist texts fail to take responsibility for the specific role that Euro-American empire (and its humanism) has played in producing health inequities and the climate crisis. Universalizing the human “narcissism” (Bennett, 2010: 25) that has produced global catastrophe ignores the fact that “only some humans are—have ever been—in charge of the world” (López, 2018: 383, responding to Bennett). Hardt and Negri (2019: 82) similarly write, “as if the species as a whole was equally responsible for the decisions that created our present predicament [. . .] a relatively small class of capitalists in the dominant countries are really responsible.” Karera’s (2019: 43, 38) note on the “historical amnesia concerning questions of culpability” suggests that posthumanism “has generally been unable to yield a sustained critique of the racist origins of global warming,” therefore “exposing the limits of its desire to rethink—to ‘revamp’ perhaps—the concept of the ‘human.’”

Building on these questions of culpability and vulnerability, Karera raises the question, *what type of future* is being imagined for post-anthropocentric times, and *for whom?* Given the unacknowledged recentering of European humanism, those who “enjoyed privilege as liberal humanist subjects will have first dibs on posthuman privilege, too” (Burton, 2017: 19; see also King, 2017; López, 2018). As several scholars have demonstrated, posthumanism not only ironically recenters and absolves the very humanism it professes to go beyond, but participates in the racializing project wherein whatever posthuman is being imagined, and whatever world it is in, retains an abandonment of many populations who have yet to be granted humanity. Karera (2019), King (2017), and Jackson (2015) argue that this abandonment of those deemed “sub-human” and “non-human” in this emerging discourse suggests that the future that posthumanism imagines is a white one—and certainly not a Black one. Whatever world we are building, whatever post-anthropocentric future we are imagining, however the “undifferentiated we” survives this crisis, the unacknowledged reality that is built into our current system is that only *some of us—*the “raceless liberal humanist subjects” will survive (López, 2018: 383; see also Karera, 2019; Mignolo and Walsh, 2018).

Embedded within this is the reality that the obsession with futurity, survivability, and a sudden realization of the possibilities that these might not be possible, is an obsession amongst white elites, who, on a population level, frankly have not had to grapple with such an existential crisis before. In fact, as Karera (2019: 33) and other scholars write at length, one has to have a “hyper-valuation of the concept life” to be caught up with the grief and loss of futurity, vitality, and health that has been so expected by and granted to white elite populations and yet systematically foreclosed for colonized populations (see also Nhemachena and Mawere, 2020; Puar, 2017). Karera (2019: 34, 51) argues that if the posthumanists get it their way, those who survive the climate crisis (who are also those who expect to, want to, feel entitled to, and so yearn to survive) will “merely recalibrate and reproduce anti-black racist practices” and sentiments that continue to foreclose the notion of long-term survival for much of the world’s populations (see also Puar, 2017). This colorblind “hyper-valuation” of life and fixation with (white) futurity presents a critical caution for rehabilitation science scholars, where we might find ourselves engaging with critical posthumanism’s notions of an affirmative panhuman community (Braidotti, 2013) and interdependent “vibrant matter” (Bennett, 2010), while paying less attention to its critiques of European humanism’s hierarchization of humans. This selective uptake risks obscuring how these hierarchies shape responses to catastrophes such as the climate crisis, as well as the genocidal military operations that contribute so forcefully to it (Hughes et al., 2022; Shaheen et al., 2024).

Learning from these scholars, one question that arises is whether posthumanism—and the Western presuppositions upon which it stands—mainly serves white, globally elite populations, thus perpetuating the violences of (settler) colonialism, racial capitalism, and white supremacy. In other words, the elite white populations who have historically benefited from the conceptualization of the European human are already benefiting from posthumanism and will continue to, lest anti-Blackness and (settler) colonialism are grappled with (Burton, 2017). In rehabilitation scholarship, the use of posthumanist theory in notions of planetary health and holistic health practice cannot account for “the mundane and persistent ways in which death and perhaps even extinction always already constitute existence for the ‘fungible’ object/being” (Karera, 2019: 46; for more critiques of Western disability empowerment, see Puar, 2017). If we take the critiques offered above seriously, we wonder, how we, as rehabilitation scientists, can continue to insist on being proponents for health, when our theorizations of the human continue to exclude certain populations and obscure the imperial production of their ongoing debilitation and impairment? Is it time to reevaluate what and whose ends we are being made to serve (Deleuze, 1990)?

## All matter matters?

The rapid and rising tide of interest in certain kinds of nonhumanism [. . .], a radically proscribed notion of who and what qualifies as ‘human’—particularly in the face of movements such as Black Lives Matter, and intellectual formations such as global south studies—must be accounted for (Lopez, 2018: 374).

The “All Lives Matter” slogan became popular among conservatives and centrists in the USA in reaction to the Black Lives Matter movement and its calls to end anti-Black racism and police violence. As many have argued, “All Lives Matter” is a reaction to calls for change and thereby insists on the status quo, while maintaining a veneer of a regard for all lives (Biko, 2018). Thereby, it reasserts the ongoing historical anti-Black sentiment where Black lives necessarily continue to be excluded from the category of “all lives” and from those who “matter.” Regalado (2021) extends this commentary on “All Lives Matters,” arguing that this sentiment has been *in the air*, influencing multiple other domains such as the recent universalizing discourses about vulnerability during the COVID-19 pandemic. The United Nations (2020) initially endorsed the slogan “we’re all in this together [. . .] The virus threatens everyone,” (Guterres, 2020, as cited in Regalado, 2021), with some government officials expressing “this virus is the great equalizer,” (Cuomo, 2020, as cited in Regalado, 2021). As Regalado (2021) argues, “the issue of systemic racism became obscured in the face of an ‘All Lives Matter’ sentiment that failed to recognize that not all lives were equally threatened” (see also Bowleg, 2020; Mein, 2020; Sandset, 2021). We wonder if posthumanism in rehabilitation science risks suffering a similar fate and serving similar ends.

While posthumanism has offered new avenues for escaping the harms of the Enlightenment’s humanism, it appears that the long and ongoing call by critical race scholars and activists to turn toward humans who have been deemed “sub-human” and “non-human” has been met with “all matter matters,” especially “more-than-human” matter. Posthumanism responds by prioritizing and granting agency to more-than-human objects rather than to racialized (in)humans, who, as Karera (2019: 46) notes, have been historically and continue to be deemed disposable (see also Puar, 2017). In other words, revitalizing matter, moving toward the “more-than-human,” and vaunting non-human objects does not guarantee that humans-deemed-objects will be valued (Puar, 2017: 26). To be clear, it is not the valorization of the more-than-human in itself that is the problem but, as Islam (2016: 122) argues, it is when done at the expense of the “human-other” that constitutes “the neo-colonial move” in posthumanism that removes “the human subaltern groups from the discursive space” and “may help neo-techno-colonizers in the act of exploitation, since their exploitation will remain invisible.” Given the profound silence on matters of racism in posthumanist rehabilitation scholarship, we wonder if it might be serving as a “get out of jail free card” for the field on matters of race, settler-colonialism, Euro-American empire, and racial capitalism (Chen, in Apter et al., 2016, referenced in Galloway and Culp, 2016). These are urgent questions that we, the authors, and we think, rehabilitation science needs to contend with.

## The desire that is posthumanism

Following the critiques offered above we wonder, what is this desire that is posthumanism? What does posthumanism want? In other words, why is it that there has been a proliferation of mainly White, Western rehabilitation scholars in the “Global North” (ourselves included) insisting for the “move beyond the human” and the “turn to matter,” often without any mention of the fact that “humanity” is still a status that has not been granted to many disenfranchised populations across the world (Agamben, 1998; De La Cadena and Blaser, 2018; Fanon, 1961; Freire, 2018; Karera, 2019; King, 2017; Mbembe, 2019; Mignolo, 2012; Mignolo and Walsh, 2018; Moten, 2013; Nhemachena and Mawere, 2020; Puar, 2017; Sharpe, 2016; Weheliye, 2014; Wilderson, 2008, 2020; Wynter, 1984, 2003)? What forces make this possible? What are the consequences? What kind of subject is afforded the comfort, capacity, and unprocessed guilt to hold all humans equally responsible for the current hellscape, and then, in a Muskian fashion, fantasize and pontificate escaping it (Hardt and Negri, 2019)? Is it merely an unfortunate coincidence that posthumanism has been particularly popular amongst White, globally elite scholars who are increasingly being urged and pressured to take responsibility and accountability for our and our ancestors’ part in historically produced systems of oppression? Perhaps not. After all, who might be driven by a desire to outsource responsibility and “authority to a place beyond the human” (Galloway, 2016) Perhaps those comforted by a universalizing politics that secures settler futurity (Tuck and Yang, 2012). Weheliye (2014: 23) notes, posthumanism “frequently appears as little more than the White liberal subject in techno-informational disguise.” As alluded to by Karera’s comments on responsibility, this politics occurs through the deferral of “any direct form of political analysis that might involve laying blame at the feet of a specific individual or set of individuals” (Buchanan, 2020: 118), thus pre-emptively absolving oneself of political critique. That posthumanism is the next “White” thing is unsurprising if we consider “the structural conditions that facilitates and renders possible the ‘symptomatic desire to abandon race’” (Karera, 2019: 44, referencing Leong, 2016: 12–13).

We contend that posthumanism has ontological value, and like Hassan (1977), Hayles (1999), and Polsky (2022), agree it accounts well for the inherent and ongoing relationality of human and non-human matter. However, as Ellis (2018: 165) argues, we must not “be tempted to conflate this restive unsettling with revolutionary insurgency, or ‘lines of flight’ with liberation, changefulness and open-endedness in and of themselves are neither an ethics nor a politics.” While ontologically it may be true that *all matter matters*, ontology is always political; assertions that “all lives” or “all matter” matters are built on the elision of the “geopolitics of racial ontology” that we have inhabited for centuries, and arguably require reparative readings rather than escapist appeals to (even immanent) truths (Puar, 2017: 55). The value of a given theoretical framework has less to do with what it *is* or even if it is “true,” and more to do with the desire that sets it in motion, and what it subsequently *does*. A hammer, for instance, can be a tool or a weapon depending on how and why it is wielded and by whom (Buchanan, 2020). Ellis (2018: 165) continues, reminding that “if the dynamic fluidity of posthumanism’s assemblage ontology promises openings for change, it cannot tell us what changes to wish for. Its materialist perspective thus helps us to recognize the more-than-human forces that populate politics, but it does not yield a materialist politics.” Therefore “it is utterly crucial not to leave these fields alone to play in their unraced genealogies” and we must insist “on an analysis of the subhuman or not quite human along with the cyborgian and the posthuman” (Puar, 2017: 26). Following the above critiques, posthumanism as a call or vision for the future perhaps has little ethical utility in rehabilitation science beyond providing a means for (some) people to escape the material history and consequences of European humanism.

## Now what?

Being present with the critiques offered in this article, we wonder: If rehabilitation science is a field that seeks to improve the health of all people who experience illness and injury, then what is our responsibility as scholars? How can we contribute important theoretical and empirical scholarship toward improving individual and population health, while being accountable to more than just White, globally elite populations already granted unfettered notions of futurity? How do we cultivate a healthier world without simply replicating the colonial and anti-Black violence of this one (Karera, 2019)? After all, most illnesses and disabilities are present among the racialized majority in the “Global South” (Connell, 2011; Livingston, 2005; Meekosha, 2011; Puar, 2017).

Before we offer any responses, we would like to pause and encourage a criticality and hesitation about any compulsion to offer solutions. When faced with critique that threatens White futurity, it is not uncommon to feel the impulse to plosively insist “so what do you propose?” However, as Jansen et al. (2021) remind us, it is this “posterizing impulse,” a kind of action bias and desire to urgently reinstate White futurity, that got us here in the first place. This impulse is deeply integrated into Western colonial society and convivial with what Shay (1957: 5) called the “myth of progress”—the colonial belief that “progress has taken place, that we are experiencing the results of past progress, and that there is a strong possibility if not inevitability that progress will continue in the future.” The “myth of progress” thus works to urgently forget and normalize the colonial past (what got us here), present (what went/is going wrong), and cultivate settler futurity—the futurity of those who expect and will be granted access to futurity (Puar, 2017: 15)—while erasing “certain kinds of subjectivities that don’t have access to futurity, in particular settler colonial subjectivities that have understood futurity as an untenable political horizon” (CLAGS: The Center for LGBTQ Studies, 2013, *Puar’s keynote address at 46:30 minutes*). The yearning for solutions—particularly those proffered within the academy—can reinforce existing structures if we are not intentional about actively refusing them.

The question about what the future looks like is a very different question from different kinds of locations and isn’t answerable from one location. [. . .] One way of thinking through a solidarity politics is to understand that in the name of futurity a lot of biopolitical violence happens on a daily basis [. . .] An alliance that understands those biopolitical violences needs to refuse [. . .] a politics of longevity that insists that one person’s access to the future is at the expense of another person’s access to the future. The question about the future is a relational one and it’s a biopolitical one and it is not something that can be collectively answered unless we take up the problem of who has access to the future and who doesn’t. (CLAGS: The Center for LGBTQ Studies, 2013, Puar’s keynote address at 46:30 minutes).

This work of refusal happens collectively in our communities, as does what we “do next,” or do instead, or keep doing, or do alongside, and so on. Perhaps this means that we White scholars stop theorizing “the next thing” (St. Pierre, 2022: 29). What we “do next” and *how* is not for the four of us to decide. Thus, in this article we will not offer any new theory of the human or direction for the field of rehabilitation science. Instead, we echo De La Cadena’s (2010) and Stengers’ call to “slow down reasoning”—“to let the composition of that which does not have a political voice (or, in some cases, does not want to have one) affect [our] analysis” (De La Cadena, 2010: 358, referencing Stengers, 2005). Perhaps it is our responsibility to slow down, pause, and consider what our scholarship and theories are doing—what and whose ends they are serving (Deleuze, 1990). The *work* this paper undertakes is not intended to be found in its conclusion or implications but in contending with the literature referenced above, what it is doing, and how it is re/working posthuman thinking. Guided by these insights, we consider the responsibility that we have to seek out, learn from, draw on, aim toward, and uplift theoretical approaches that can:

1) Participate in the crucial project of articulating the intersections between race, racism, health, and debility (Puar, 2017; Watego et al., 2021).
2) Address how a lack of progress in health equity is linked to a failure to confront the pasts and presents of (settler) colonialism, imperialism, and chattel slavery.
3) Show how modern (colonial) health systems are shaped by and often perpetuate these system’s exclusion and exploitation, which live on in structural health inequities.
4) Contribute to knowledge that does not recolonize and perpetuate harm (Williams and Marlin, 2022).

To do this, we echo what the authors we have referenced in this essay have been calling for, and invite fellow rehabilitation science scholars who encounter posthumanism (or any Eurocentric theories of the human) to consider the following:

1) If posthumanism appeals to you, as it has to us, pause and take a moment to reflect on why that might be and be with the critiques put forward by those referenced in this article.
2) Pause, and proceed with great caution. As Weheliye (2014: 48) urges, “remain cautious [. . .] about the complete disavowal of subjectivity in theoretical discourse, because within the context of the Anglo-American academy more often than not an insistence on transcending limited notions of the subject or identity leads to the neglect of race as a critical category.” If history has taught us anything, it is to be critical of those who, with one hand, proffer universal truths about what it means to be human, because invariably, behind the back the other hand has already decided which populations are not-quite-human and therefore disposable.
3) Instead of following the taken-for-granted obviousness and “liberating potentials” of posthumanism, pay attention to, read, cite, listen to, and learn from scholars that have not only long contested European humanism and its harms, but have offered alternative expression of the “human” that have “deep commitments to those entities that are instrumentally denied humanity in order for it to be sustained” (Puar, 2017: 29).
4) Refuse to lose sight of those for whom posthumanism does “not necessarily hold any emancipating value” (Karera, 2019: 34).

Colonialism, empire, and racial capitalism contribute significantly to health inequity through dehumanization, neglect, exploitation, and direct violence. If we are to attend to these urgent matters and contribute to the health equity goals that rehabilitation science purports to espouse, then we must carefully consider our theories, especially, or theories of the human.

Understand yourself, white man. You are the savages. Yes, it is you who have always been uncivilized. Civilize yourself.

-Stokely Carmichael
